# Systemic autoinflammatory disease following COVID-19 mRNA vaccine: a severe and rare clinical presentation

**DOI:** 10.1007/s11739-025-03965-9

**Published:** 2025-05-15

**Authors:** Federica Maiolini, Eleonora Bassanello, Jacopo Croce, Elisa Tinazzi, Simonetta Friso

**Affiliations:** Department of Medicine, Unit of Internal Medicine B, University of Verona School of Medicine, Policlinico G.B. Rossi, Piazzale L.A. Scuro, 10, 37131 Verona, Italy

## Case presentation

### Case 1

A 48-year-old woman, without relevant medical records, was referred to our Internal Medicine Unit for persistent elevated fever (over 38.5 °C) that had lasted for about 1 month and started the day after she had received the second shot of COVID-19 mRNA vaccine.

Upon admission, the patient denied accompanying symptoms and, at physical examination, no significant pathological signs were detected. Blood tests showed only increased C-reactive protein (CRP) levels (Table 1). In the differential diagnostic process, we indeed considered an infective, malignant and/or autoimmune etiology of her febrile status. Multiple blood and urine cultures were performed that resulted sterile as negative for procalcitonin, along with the serology for viruses and autoimmune pattern tests. Total body scan and bone marrow biopsy did not show any pathological signs. She was successfully treated with prednisone 25 mg die ex-juvantibus. After 4 months, she experienced fever relapse during decalage of steroid therapy.

**Table 1:** Synopsis of laboratory parameters at admission and during hospitalization in Case 1 and Case 2

	Normal values	Case 1			Case 2	
		1 st admission	1 st episode of MAS	2nd episode of MAS	2nd admission at ED	At our unit
Hb (g/dL)	12–15 (case 1, female) 13.5–17 (case 2, male)	10.9	6.5	6.1	12.2	9.7
WBC (n/mmc)	4,300–100,00	5620	260	140	12,970	20,660
PLTs (n/mmc)	150,000–400,000	328,000	34,000	41,000	105,000	130,000
CRP (mg/L)	< 5	79	10	88	287	317
Ferritin (µg/L)	30–300	72	9,237	150,000		1,292
AST (U/L)	5–50	22	422	44		99
ALT (U/L)	6–50	16	388	45	79	176
Creatinine (µmol/l)	0.59–1.29 52–114	0.76 67.2	4.43 391	2.29 202.4	1.10 97.2	1.67 147.6
Albumin (g/L)	35–50	39	24.9	26		28.5
Fibrinogen (g/L)	2–4	5.76	0.52	1.03		10.9
Procalcitonin (ng/ml)	0.05–0.5	0.08	0.7	1	0.05	0.05
Quantiferon test	Negative	Negative			Negative	
HIV	Negative	Negative			Negative	
Parvovirus B19 serology	Negative	Negative			Negative	
Cytomegalovirus DNA	Negative	Negative			Negative	
EBV DNA	Negative	Negative			Negative	
HBsAg	Negative	Negative			Negative	
Anti HBs Ab	Negative	Negative			Negative	
Anti HBc Ab tot	Negative	Negative			Negative	
Anti HCV ab	Negative	Negative			Negative	
Toxoplasmosis serology	Negative	Negative			Negative	
Leishmania serology	Negative	Negative			Negative	
Borrelia serology	Negative	Negative			Negative	
Blood culture 2 sets	Negative	Negative			Negative	
Beta D-Glucan	Negative	Negative	Negative	Positive	Negative	
Galactomannan	Negative	Negative	Negative	Negative	Negative	
RF (KIU/L)	< 14	12			10	
ANA (ratio)	< 1/80	< 1/80			< 1/80	
ENA (UA)	0–20	3			5	
Anti-ds-DNA (CLIA UI/ml)	0–27	23			12	
ASMA	< 1/80	< 1/80			< 1/80	
AMA	< 1/80	< 1/80			< 1/80	
p-ANCA (ratio)	< 1/80	< 1/80			< 1/80	
c-ANCA (ratio)	< 1/80	< 1/80			< 1/80	
C3 (mg/dl)	90–180	100			150	
C4 (mg/dl)	20–50	40			50	

*WBC* white blood cells, *Hb* hemoglobin, *PLTs* platelets, *CRP* C-reactive protein, *ALT* alanine aminotransferase, *AST* aspartate aminotransferase, *HIV* human-acquired immunodeficiency virus, *EBV* Epstein–Barr virus, *anti HbsAg* antibodies anti-Hepatitis B antigen, *anti HBc Ab* anti-Hepatitis virus core antibodies, *RF* rheumatoid factor, *ANA* antinuclear antibodies, *ENA* extractable nuclear antigen, *anti ds DNA* anti-double-strand DNA antibodies, *ASMA* anti-smooth-muscle antibodies*, AMA* anti-mitochondrial antibody*, p-ANCA* anti-myeloperoxidase (MPO) antineutrophil cytoplasm antibodies, *c-ANCA* anti-proteinase-3 (PR3) antineutrophil cytoplasm antibodies, *C3* complement fraction 3, *C4* complement fraction 4

### Case 2

A 56-year-old man complained about the onset of elevated fever (over 39 °C) a few hours after receiving the booster dose of COVID-19 mRNA vaccine. After 7 days of persistent fever, he presented to the emergency department (ED) where first-line laboratory tests showed elevated CRP with a complete blood count and transaminase levels within the normal range. He was discharged from ED with paracetamol. Due to persistence of high pyrexia and intense sore throat, he returned to the ED where he underwent laboratory tests that showed systemic inflammation associated to mild anemia, thrombocytopenia, and increased transaminases (Table 1).

## Further investigation and differential diagnosis

### Case 1

During steroid therapy decalage, we observed a relapse of fever that was accompanied by symptoms such as pharyngodynia, arthralgias, and signs such as skin rash, splenomegaly, and lymphadenopathy. The patient was then re-admitted to our Unit where microbiologic and autoimmune screening was repeated and all resulted negative. Laboratory tests showed leukocytosis with neutropenia, CRP 80 mg/l, ferritin 1500 μg/l. An echocardiography was negative for valvular vegetations. A positron emission tomography with 2-deoxy-2 fluoro D glucose integrated with CT was then performed and showed a diffuse uptake of 18-FDG by the bone marrow and the spleen**.** The patient was then diagnosed with adult onset still disease (AOSD) according to Yamaguchi’s criteria and treated with prednisone 1 mg/kg die in association with subcutaneous (sc) anakinra 100 mg twice a day with initial benefit so that she was discharged home. Because of the onset of refractory migraine that was interpreted as an anakinra collateral effect, she was switched to sc tocilizumab (162 mg every 7 days).

**Fig. 1:**
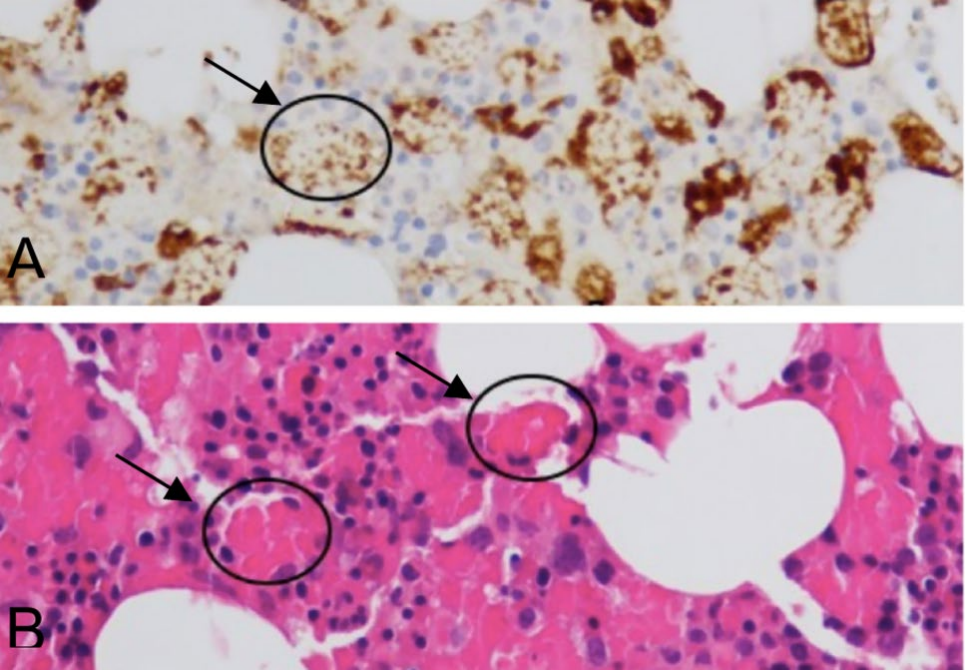
Histological and immunohistochemical findings in bone marrow biopsy from Case 1 patient. Panel **A** CD68 positive histiocytes are displayed (immunohistochemical staining; X40). Panel **B** Phagocytosis of red blood cells is shown (arrow), (hematoxylin–eosin staining; X40)

Three weeks later, she reported again a relapse of high fever, intense arthralgias, and skin rash so she was re-admitted to our Unit. The laboratory data showed pancytopenia and increased transaminases, severe hyperferritinemia (Table 1). A sepsis and a SARS-CoV-2 infection were excluded, but a progressive multi-organ failure was observed needing non-invasive ventilation, continuous renal replacement therapy, red blood cells and platelets transfusions, and fibrinogen and albumin supplementation. Clinical diagnosis of macrophage activation syndrome (MAS) was supported by the bone marrow biopsy, showing the presence of hemophagocytosis (Fig. 1). Treatment with intravenous (iv) anakinra 600 mg die, dexamethasone (16 mg die) and etoposide (100 mg) was started. MAS clinical remission was achieved for 1 month. Systemic steroid therapy was gradually reduced, and etoposide was withdrawn. Anakinra was tapered (200 mg die iv).

The patient experienced a MAS relapse in concomitance with SARS-CoV-2 infection, ferritinemia was 150.000 μg/l, and the treatment with dexamethasone and etoposide was administered again. The patient unfortunately developed a disseminated lung candidiasis which caused the exitus.

### Case 2

He was admitted to our Unit where we started a differential diagnosis process. About a possible infectious etiology, multiple blood cultures, viral serological tests were negative. An echocardiography, a CT scan of chest and abdomen did not reveal any signs of infection or solid neoplasia. The bone marrow biopsy was also negative. Tests for autoimmune diseases such as ANA, ENA, anti nDNA antibodies, RF were negative. In the meantime, laboratory blood test showed increased CRP and transaminases, hyperferritinemia, hypoalbuminemia, and acute kidney injury (Table 1).

According to Yamaguchi’s criteria, we diagnosed AOSD, and an early treatment with dexamethasone 8 mg bid and sc anakinra 100 mg bid was started with optimal response. No relapse of fever during steroid tapering was observed, but the patient complained of arthralgia, so sc methotrexate 12.5 mg was successfully added every 7 days. After 24 months of follow-up, no relapse was registered and the steroid was withdrawn.

## Discussion

SARS-CoV-2 pandemic infection caused thousands of deaths and the vaccination was the life-saving tool but some adverse effects have been also described among patients such as redness at the injection site, fever, fatigue, headache, chills, vomiting, and urticaria. Although rare, some cases of systemic hyperinflammation after vaccination have been reported, highlighting the need for a precocious identification and treatment of severe clinical manifestation [1].

AOSD is a rare autoinflammatory disorder of unknown etiology characterized by a severe hyperinflammatory state. The characteristic symptoms of the AOSD are spiking fever, hyperferritinemia, arthritis, evanescent rash, sore throat, hepatomegaly, splenomegaly, lymphadenopathy, and serositis [2].

A potential life-threating complication of AOSD, reported in up to 23% of cases, is MAS, an acute systemic inflammatory syndrome, which is characterized by a massive cytokine “storm”, whose occurrence can be related to a poor prognosis [2].

The etiology of AOSD is still unclear, it is a multifactorial disease: in case of genetically susceptible patients, an immunogenic stimulus, such as an infection (viral or bacterial), a solid or hematological cancer, could trigger a hyperinflammatory state [3].

We described here two cases of AOSD that developed after vaccination with mRNA COVID-19 vaccine. The pathogenetic mechanism underlying the occurrence of an autoimmune or autoinflammatory disease after a vaccination has not been yet clarified: a viral infection can trigger autoimmune or autoinflammatory diseases through the mechanisms of the molecular mimicry and the bystander activation [4]. The former represents a shared immunological epitope with a pathogen and the host, the latter is the activation of an antigen-presenting cell which can activate a pre-primed autoreactive T cell. In our first case, the relapse of MAS has been probably driven by SARS-CoV-2 infection itself [5]. The same mechanisms of cross reactivity could be elicited by a vaccination.

In the case of persistent elevated fever, especially in the appearance of an evolving hyperinflammatory state, the clinician should precociously consider the diagnostic hypothesis of an autoinflammatory syndrome which could also evolve into MAS, a rare and rapidly progressing fatal complication which, therefore, needs to be intercepted early, even if it is associated with significant morbidity and mortality.

## Data Availability

The data that support the findings of this study are available from the corresponding author upon reasonable request.
